# Urinary Biomarkers for Phthalates Associated with Asthma in Norwegian Children

**DOI:** 10.1289/ehp.1205256

**Published:** 2012-11-16

**Authors:** Randi J. Bertelsen, Karin C. Lødrup Carlsen, Antonia M. Calafat, Jane A. Hoppin, Geir Håland, Petter Mowinckel, Kai-Håkon Carlsen, Martinus Løvik

**Affiliations:** 1 Department of Food, Water and Cosmetics, Norwegian Institute of Public Health, Oslo, Norway; 2 Epidemiology Branch, National Institute of Environmental Health Sciences, National Institutes of Health, Department of Health and Human Services, Research Triangle Park, North Carolina, USA; 3 Department of Pediatrics, Oslo University Hospital, Oslo, Norway; 4 Faculty of Medicine, University of Oslo, Oslo, Norway; 5 National Center for Environmental Health, Centers for Disease Control and Prevention, Atlanta, Georgia, USA

**Keywords:** asthma, biomarkers, children, endocrine disruptors, phthalates

## Abstract

Background: High-molecular-weight phthalates in indoor dust have been associated with asthma in children, but few studies have evaluated phthalate biomarkers in association with respiratory outcomes.

Objectives: We explored the association between urinary concentrations of phthalate metabolites and current asthma.

Methods: In a cross-sectional analysis, 11 metabolites of 8 phthalates [including four metabolites of di(2-ethylhexyl) phthalate] were measured in one first morning void collected from 2001 through 2004 from 623 10-year-old Norwegian children. Logistic regression models controlling for urine specific gravity, sex, parental asthma, and income were used to estimate associations between current asthma and phthalate metabolite concentrations by quartiles or as log_10_-transformed variables.

Results: Current asthma was associated with both mono(carboxyoctyl) phthalate (MCOP) and mono(carboxynonyl) phthalate (MCNP), although the association was limited to those in the highest quartile of these chemicals. The adjusted odds ratio (aOR) for current asthma was 1.9 (95% CI: 1.0, 3.3) for the highest MCOP quartile compared with the lowest quartile, and 1.3 (95% CI: 0.98, 1.7) for an interquartile-range increase. The aOR for current asthma was 2.2 (95% CI: 1.2, 4.0) for the highest MCNP quartile and 1.3 (95% CI: 1.0, 1.7) for an interquartile-range increase. The other phthalate metabolites were not associated with current asthma.

Conclusions: Current asthma was associated with the highest quartiles of MCOP and MCNP, metabolites of two high molecular weight phthalates, diisononyl phthalate and diisodecyl phthalate, respectively. Given the short biological half-life of the phthalates and the cross-sectional design, our findings should be interpreted cautiously.

Phthalates are widely used as plasticizers in consumer products such as building materials, toys, food packaging, cosmetics, and medical devices (Schettler 2006). The length of the alkyl chains gives rise to different molecular weights and functionality between the different types of phthalates. There is no covalent bond between phthalates and the plastics in which they are mixed, and phthalates are therefore easily released into the environment. Human exposure occurs through ingestion, inhalation, and dermal contact, with contaminated food considered to be the most important source of exposure to high-molecular weight phthalates (high-MWP; metabolites > 250 Da) for the general population (Schettler 2006). The use of personal care products may increase exposure to low-MWP (Just et al. 2010; Silva et al. 2004).

Phthalate metabolite concentrations in urine represent a measure of exposure from multiple sources and routes. The only study that has reported on the phthalate burden in a Norwegian population compared concentrations of phthalate metabolites in pregnant women from Norway, the Netherlands, and the United States and found comparable levels among these three groups of women, with estimated average daily intakes below the recommended maximum intake (Ye et al. 2009). Phthalates have also been found in abundance in indoor air particulate matter in Norwegian dwellings and schools (Rakkestad et al. 2007), but so far no data on the body burden of phthalates in children in Norway are available.

In the last decade, several studies have reported associations between phthalate exposure and asthma and allergic disease (Jaakkola and Knight 2008). The findings differ among the phthalates, but most often the heavier phthalate metabolites have had impact on allergic disease development in animal models (Kimber and Dearman 2010). In *in vitro* studies, the monoester metabolite of di(2-ethylhexyl) phthalate (DEHP), mono(2-ethylhexyl) phthalate (MEHP), induced a release of inflammatory mediators from lung cells, including tumor necrosis factor α (Rakkestad et al. 2010), a pro-inflammatory cytokine that has been implicated in many aspects of the airway pathology of asthma. Several phthalates, including diisodecyl phthalate (DIDP) and phthalate metabolites such as MEHP, can activate peroxisome proliferator-activated receptors that participate in a range of cellular processes including positive and negative regulation of inflammation (Cho et al. 2008; Lovekamp-Swan and Davis 2003). In epidemiological studies, asthma and allergic diseases in children have been associated with house dust concentrations of several high-MWP that are often used in polyvinyl chloride (PVC) flooring (Bornehag et al. 2004; Kolarik et al. 2008). An early follow-up investigation of children from the same birth cohort evaluated in the present study reported that DEHP was the major phthalate in house dust (Oie et al. 1997) and that bronchial obstruction was associated with phthalate-containing PVC flooring and plastic surface materials at home (Jaakkola et al. 1999). There are few reports on associations between biomarkers of phthalate exposure and respiratory outcomes, although one study reported an inverse association between urinary concentrations of monoethyl phthalate (MEP), a metabolite of diethyl phthalate (DEP), and lung function in adult men (Hoppin et al. 2004).

The aim of the present study was to explore the association between urinary concentrations of phthalate metabolites and current asthma in children.

## Methods

*Design and subjects*. The present cross-sectional study includes 10-year-old children from the Environment and Childhood Asthma (ECA) study in Oslo, Norway, which is described in detail elsewhere (Lødrup Carlsen 2002). Briefly, this birth cohort included 3,754 healthy infants born in Oslo during a 15-month period beginning 1 January 1992. The ECA study design involved clinical investigations of two main study populations within the birth cohort. Lung function was obtained in 802 infants shortly after birth (Lødrup Carlsen et al. 1994). At 2 years of age, a nested case–control study aimed to include all children from the initial birth cohort who had recurrent (> 1) or persistent (> 4 weeks) doctor-confirmed bronchial obstruction, along with age-matched controls (Lødrup Carlsen 2002). Children with lung function measured at birth or who had participated in the case–control study at 2 years of age were invited to a 10-year follow-up; 1,019 (84%) children participated in the investigation [see Supplemental Material, Figure S1 (http://dx.doi.org/10.1289/ehp.1205256)].

Of the 1,019 children who participated in the 10-year follow-up, funds were available to analyze urine specimens for 623 children. Subjects who had current asthma at 10 years were preferentially sampled [see Supplemental Material, Figure S1 (http://dx.doi.org/10.1289/ehp.1205256)], yielding a higher prevalence of current asthma (21%) than in the 396 children who participated in the ECA 10-year follow-up but were not included in the present study (7%) (see Supplemental Material, Table S1). The 623 children in the present study had a slightly lower median age (10.7 vs. 10.8 years), a higher prevalence of atopic eczema (23% vs. 18%), a lower prevalence of allergic sensitization [a positive skin prick test or elevated serum-specific IgE (≥ 0.35 kU/L) for at least one of the following allergens tested: house dust mites (*Dematophagoides pteronyssinus* and *D. farinae*), cat, dog, rabbit, German cockroach, birch, timothy, and mugworth pollen, *Cladosporium, Alternaria*, egg white, milk, peanut and codfish; 35% vs. 43%] and were less likely to be firstborn (49% vs. 56%) than the 396 excluded children. The included children were comparable to the remainder of the original study cohort at birth with regard to most characteristics, but were less likely to be first-born (49% vs. 56%) and to be in a middle-income family (see Supplemental Material, Table S2).

The 10-year follow-up of the ECA study was approved by the Regional Committee for Medical Research Ethics and the Norwegian Data Inspectorate, and the biobank was registered in the Norwegian Biobank Registry (Oslo). The parents gave written informed consent for their child’s participation in the study. The involvement of the Centers for Disease Control and Prevention (CDC) laboratory was determined not to constitute engagement in human subjects research.

*Clinical examination and outcome definition*. The 10-year follow-up was performed between September 2001 and December 2004, and included a detailed parental structured interview, blood tests, skin prick tests (SPT) for allergic sensitization, spirometry, first morning urine sampling, and clinical examinations.

Current asthma (Lødrup Carlsen et al. 2006) was defined by a history of asthma reported by the parent plus at least one of the following: reported dyspnea, chest tightness, and/or wheezing during the previous 12 months; reported use of asthma medication during the previous 12 months; or a positive exercise challenge test (a ≥ 10% decrease in forced expiratory volume in 1 sec compared with baseline, performed 2–7 days after the first visit).

*Urine collection and analyses*. The participants’ first morning voids, collected at home in commercial polypropylene specimen collection containers, were aliquoted and frozen at –80ºC in 2 mL polypropylene tubes until they were shipped on dry ice to the CDC (Atlanta, GA, USA) for analysis. Three low-MWP metabolites [molecular weight < 250 Da; MEP, mono-*n*-butyl phthalate (MnBP), and monoisobutyl phthalate (MiBP)] and eight high-MWP metabolites [molecular weight > 250 Da; monobenzyl phthalate (MBzP), mono(3-carboxypropyl) phthalate (MCPP), monocarboxyoctyl phthalate (MCOP), and monocarboxynonyl phthalate (MCNP), including four metabolites from DEHP: MEHP, mono(2-ethyl-5-oxohexyl) phthalate (MEOHP), mono(2-ethyl-5-hydroxyhexyl) phthalate (MEHHP), and mono(2-ethyl-5-carboxypentyl) phthalate (MECPP)], were measured in the urine by online solid phase extraction coupled with high-performance liquid chromatography with isotope-dilution tandem mass spectrometry using a modification of the method reported by Silva et al. (2007). We multiplied the reported MEP and MBzP concentrations by 0.66 and 0.72, respectively, to correct for the inadequate purity of the analytic standards used. The lower-than-expected purity of the standards does not affect the performance of the analytical methods (CDC 2012). After analyses, the urine samples were shipped on dry ice to the Norwegian Institute of Public Health. Urine specific gravity (SG) was measured by a handheld refractometer (Atago Urine Specific Gravity Refractometer PAL 10-S; ATAGO USA, Inc., Bellevue, WA, USA) after thawing and thorough mixing of the sample with a vortex shaker. The refractometer was calibrated with distilled water between each measurement.

*Statistical analyses*. SG-corrected urinary concentrations of phthalate metabolites were compared between girls and boys by analysis of variance (ANOVA). SG-corrected metabolite concentrations were calculated by the formula *P*_c_ = *P*[(1.024-1)/(*SG*-1)], where *P*_c_ is the SG-corrected phthalate metabolite concentration (micrograms per liter), *P* is the observed phthalate metabolite concentration, 1.024 is the median SG value in the study population, and *SG* is the specific gravity of the individual urine sample. For phthalate metabolite concentrations below the limit of detection (LOD), we used an imputed value equal to LOD/_√_^–^2. Correlations between the individual phthalate metabolites were evaluated using Spearman rank correlation coefficients (*r*_S_).

For current asthma, we fit logistic regression models by quartile concentration categories of the metabolites (lowest concentration category as reference) and a second model with phthalate metabolites as continuous variables (log_10_-transformed). SG was used as a covariate in the models. We modeled each of the individual phthalate metabolites separately, except for the DEHP metabolites (MEHP, MEOHP, MEHHP, and MECCP), which were combined into their micromolar sum (sum of the individual metabolites concentrations in micrograms per liter divided by the molecular weight of the metabolites) (ΣDEHP). We fitted additional models for the micromolar sums of high-molecular weight (Σhigh-MWP) and low-molecular weight (Σlow-MWP) metabolites. To test for trend across quartiles, we fitted the median quartile concentration values as a continuous variable in the logistic regression model.

Covariates were selected based on a directed acyclic graph [see Supplemental Material, Figure S2 (http://dx.doi.org/10.1289/ehp.1205256)] that included the following variables: sex, body mass index (BMI; age and sex adjusted), allergic sensitization in the child, parental smoking at home [between the school age of the child (6–7 years) and the 10-year follow-up], parental asthma (at child’s birth), maternal education (at child’s birth), and household income (at the 10-year follow-up). The minimal adjustment set for a total effect of phthalates on current asthma was sex, parental asthma, and household income. To examine the role of allergic sensitization in the association between phthalate metabolites and asthma, we fitted additional models after stratification by allergic sensitization. Finally, we tested for modification effects by sex, by adding interaction terms to the models. *p*-Values ≤ 0.05 were considered statistically significant. All statistical analyses were performed with Statistical Package for Social Sciences (SPSS version 19.0; IBM Inc., Chicago, IL, USA).

## Results

The present study is based on data and urinary concentrations of phthalate metabolites from 623 children (294 girls and 329 boys). The median age was 10.7 years (range, 8.8–12.5) at the time of urine collection and asthma status evaluation. Twenty-one percent met our definition of current asthma (82 boys and 46 girls), and asthma status was unavailable for one boy. The participating boys were more likely to have current rhinitis and to be allergically sensitized than girls, but they did not differ with regard to the presence of current atopic eczema or parental characteristics (Table 1).

**Table 1:** Subject and parental characteristics for all participants, and for girls and boys separately (%).

Characteristic	All (n = 623)	Girls (n = 294)	Boys (n = 329)
Subjects
BMI ≥ 85th percentilea	16	17	15
Firstborn	49	47	50
Asthma, ever	33	28*	38
Current asthma	21	16*	25
Either SPT- or sIgE-positive	35	28*	41
Current rhinitisb	26	18*	27
Current atopic eczemab	23	23	24
Parents
Asthma	14	13	15
Asthma and/or rhinitis	36
Maternal education (years)
≤ 12	47	50	45
13–16	31	30	33
≥ 17	22	20	22
Annual household income (1,000 NOK)
< 350	13	12	14
> 350–560	28	28	27
> 560–750	30	85	31
> 750	30	31	28
Parental smoking at home	38	41	36
NOK, Norwegian krone. Information was missing for firstborn (n = 31), asthma (ever and current) and rhinitis (n = 1), for SPT and sIgE combined (n = 21), current atopic eczema (n = 2), maternal education (n = 2), income (n = 6), parental smoking (n = 3). aBMI, age and sex adjusted. bSymptoms occurring during the last 12 months. *p < 0.01 for difference between girls and boys.			

*Body burden of phthalates*. Detectable concentrations were found for all phthalate metabolites except MCNP, MCPP, and MEHP, which were below the LOD in < 4% of the urine samples (Table 2). Urine SG was higher in boys (1.024; range, 1.004–1.039) than in girls (1.023; range, 1.006–1.036, *p* < 0.001). Strong correlations were observed between the urinary concentrations of MCOP and MCNP (*r*_S_ = 0.75, *p* < 0.001) and between the oxidative metabolites of DEHP (MECPP, MEOHP, and MEHHP, all *r*_S_ > 0.9, *p* < 0.001) [see Supplemental Material, Table S3 (http://dx.doi.org/10.1289/ehp.1205256)].

**Table 2:** Distribution of phthalate metabolites concentrations in urine for 623 Norwegian children (with uncorrected and SG-corrected values).

Phthalate metabolite (µg/L)	LOD	n < LOD	GM	Minimum	Percentile					Maximum
					5th	25th	50th	75th	95th	
								
MEP	0.4	0	60.9	8.5	16.7	32.6	56.7	94.4	360.2	6,006
SG-corrected			64.4	7.0	19.9	35.3	56.3	101.1	320.2	5,339
MnBP	0.4	0	138.8	10.4	50.9	93.6	138.0	209.0	377.2	1,480
SG-corrected			146.7	10.0	58.7	101.1	141.0	215.2	378.9	1,429
MiBP	0.2	0	53.6	3.1	15.6	31.4	49.2	88.4	231.0	1,480
SG-corrected			57.0	6.3	18.8	34.6	50.1	90.5	239.6	1,480
MBzP	0.2	0	30.7	2.1	8.2	16.9	29.3	52.9	128.7	6,710
SG-corrected			32.5	1.6	9.7	18.7	30.8	53.2	135.3	5,195
MCPP	0.2	23	7.8	LOD	2.6	4.6	7.5	12.8	26.2	384.0
SG-corrected			8.2	LOD	3.6	5.4	7.9	12.5	24.7	307.2
MEHP	1.2	21	7.8	LOD	2.0	4.6	7.8	12.9	30.3	203.0
SG-corrected			8.2	LOD	2.4	5.0	8.2	12.5	28.7	211.8
MEOHP	0.7	0	49.8	2.4	15.0	31.2	49.7	75.3	183.0	1,220
SG-corrected			52.6	3.2	19.6	36.1	51.0	75.3	159.7	1,273
MEHHP	0.7	0	78.6	4.0	23.4	48.7	76.6	120.0	293.6	1,770
SG-corrected			83.1	5.3	31.9	56.9	79.5	116.4	271.0	1,847
MECPP	0.6	0	101.2	11.3	29.1	62.2	98.2	162.0	380.4	3,520
SG-corrected			106.9	11.9	38.9	71.7	105.0	153.2	343.2	3,673
MCOP	0.2	0	6.1	0.8	1.7	3.5	6.0	10.2	21.2	474.0
SG-corrected			6.5	0.6	2.1	3.8	6.2	10.2	21.9	421.3
MCNP	0.2	23	2.2	LOD	0.6	1.4	2.1	3.5	8.3	168.0
SG-corrected			2.3	LOD	0.7	1.5	2.2	3.6	7.8	162.8
Phthalate sums (µmol/L)										
∑low-MWPa			1.32	0.11	0.46	0.86	1.27	1.97	4.28	33.1
SG-corrected			1.40	0.11	0.56	0.92	1.30	2.02	4.26	29.5
∑high-MWPb			1.05	0.14	0.36	0.66	1.01	1.58	3.68	33.1
SG-corrected			1.10	0.11	0.45	0.76	1.06	1.51	3.17	29.0
∑DEHPc			0.81	0.08	0.26	0.50	0.79	1.23	2.86	22.4
SG-corrected			0.85	0.10	0.34	0.58	0.82	1.18	2.69	23.3
GM, geometric mean. a∑low-MWP: MEP, MnBP, and MiBP. b∑high-MWP: MBzP, MCNP, MCOP, MCPP, MEHP, MECPP, MEHHP, and MEOHP. c∑DEHP: MEHP, MECPP, MEHHP, and MEOHP.										

Physical and demographic characteristics were associated with phthalate metabolite concentrations (data not shown). The concentrations of MBzP were higher in children with BMI ≥ 85th percentile, and in children of parents in the lowest household income category (≤ 299,000 Norwegian krone) and with maternal education ≤ 12 years. MEP concentrations were higher in children of mothers with ≤ 12 years of education, and higher in children with parents reporting smoking at home compared with other children [geometric mean (GM) = 74 µg/L; 95% CI: 65, 84; and GM = 60 µg/L; 95% CI: 55, 65, respectively, *p* = 0.004].

**Figure 1:**
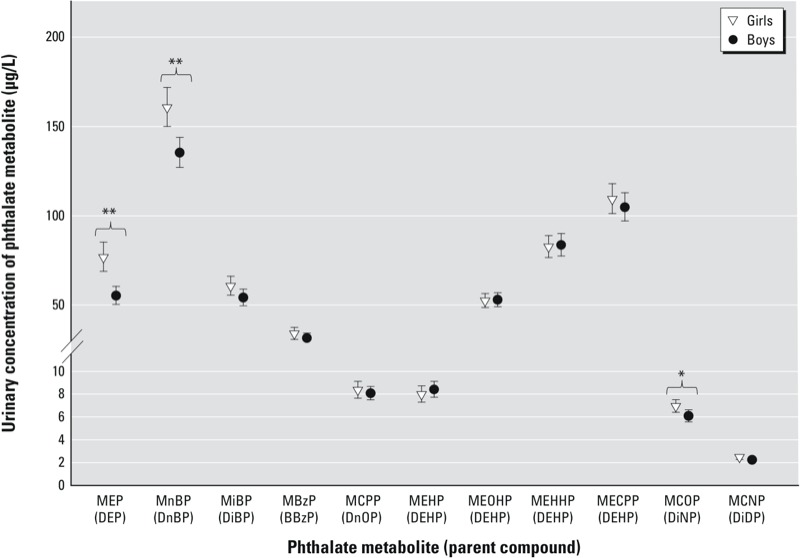
SG-adjusted phthalate metabolite concentrations (µg/L) in girls and boys. Values are GM with error bars for 95% CIs. *p < 0.05. **p< 0.001.

Compared with boys, girls had higher urinary concentrations of the low-MWP metabolites MnBP (*p* < 0.001), MEP (*p* < 0.001), and MiBP (*p* = 0.07) and of the high-MWP metabolite MCOP (*p* = 0.03) [Figure 1; see also Supplemental Material, Table S4 (http://dx.doi.org/10.1289/ehp.1205256)].

*Phthalates and asthma*. We observed statistically significant associations between the highest quartiles of MCNP and MCOP (in the adjusted analyses only) and current asthma (Table 3). The adjusted odds ratios (aORs) of current asthma increased with increasing concentration of MCOP per quartile (*p*_trend_ = 0.02) and with increasing concentration of MCNP (*p*_trend_ = 0.006) (Table 3). Girls had higher urinary concentrations of MCOP than boys (Figure 1), but there was no statistically significant modification by sex with MCOP on current asthma (*p*_interaction_ > 0.05). Phthalates were not associated with allergic sensitization (data not shown). When we stratified current asthma by allergic sensitization, the estimates for current asthma were similar for children without and with allergic sensitization, except for MCNP, which was associated with asthma among 392 children without allergic sensitization (aOR = 1.6; 95% CI: 1.1, 2.2) but not among 210 children with allergic sensitization (aOR = 1.1; 95% CI: 0.77, 1.15) [see Supplemental Material, Table S5 (http://dx.doi.org/10.1289/ehp.1205256)].

**Table 3:** Crude and adjusted odds ratios of current asthma per concentration quartile and per log10 IQR increase in MEP, MnBP, MiBP, MBzP, MCPP, MCOP, and MCNP.

Phthalate metabolite	Concentration (µg/L)	Noncases (n)	Cases (n)	Crude OR (95% CI)	aORa (95% CI)
MEP in quartiles	≤ 32.6 (ref)	119	36	1	1
	> 32.6–56.7	125	31	0.82 (0.47, 1.4)	0.97 (0.55, 1.7)
	> 56.7–94.4	127	29	0.76 (0.43, 1.4)	0.85 (0.47, 1.6)
	> 94.4	123	32	0.61 (0.50, 1.5)	0.99 (0.55, 1.8)
MEP per log10 IQR		494	128	0.82 (0.33, 2.1)	0.98 (0.39, 2.5)
MnBP in quartiles	≤ 93.6 (ref)	123	33	1	1
	> 93.6–138	124	34	1.0 (0.60, 1.8)	1.2 (0.65, 2.0)
	> 138–209	123	32	0.99 (0.56, 1.8)	1.1 (0.62, 2.0)
	> 209	124	29	0.90 (0.49, 1.6)	0.96 (0.51, 1.8)
MnBP per log10 IQR		494	128	0.83 (0.63, 1.1)	0.85 (0.64, 1.1)
MiBP in quartiles	≤ 31.4 (ref)	130	28	1	1
	> 31.4–49.2	121	33	1.3 (0.75, 2.4)	1.3 (0.74, 2.4)
	> 49.2–88.4	123	32	1.3 (0.72, 2.4)	1.4 (0.73, 2.5)
	> 88.4	120	35	1.5 (0.82, 2.7)	1.5 (0.80, 2.7)
MiBP per log10 IQR		494	128	1.1 (0.88, 1.5)	1.1 (0.87, 1.5)
MBzP In quartiles	≤ 16.9 (ref)	121	34	1	1
	> 16.9–29.2	130	25	0.70 (0.39, 1.3)	0.70 (0.39, 1.3)
	> 29.2–52.9	128	27	0.79 (0.44, 1.4)	0.83 (0.45, 1.5)
	> 52.9	115	42	1.4 (0.81, 2.4)	1.3 (0.75, 2.4)
MBzP per log10 IQR		494	128	1.2 (0.91, 1.5)	1.2 (0.88, 1.5)
MCPP in quartiles	≤ 4.6 (ref)	121	36	1	1
	> 4.6–7.5	132	23	0.61 (0.34, 1.1)	0.60 (0.33, 1.1)
	> 7.5–12.8	124	33	0.95 (0.54, 1.7)	1.0 (0.57, 1.9)
	> 12.8	117	36	1.1 (0.62, 2.1)	1.2 (0.63, 2.1)
MCPP per log10 IQR		494	128	0.99 (0.75, 1.3)	0.99 (0.74, 1.3)
MCOP in quartiles	≤ 3.5 (ref)	127	30	1	1
	> 3.5–6.0	131	27	0.93 (0.52, 1.7)	1.0 (0.60, 1.9)
	> 6.0–10.2	123	31	1.2 (0.65, 2.1)	1.2 (0.67, 2.3)
	> 10.2	113	40	1.7 (0.94, 3.0)	1.9 (1.0, 3.3)
MCOP per log10 IQR		494	128	1.3 (0.96, 1.6)	1.3 (0.98, 1.7)
MCNP in quartiles	≤ 1.4 (ref)	145	29	1	1
	> 1.4–2.1	113	26	1.2 (0.7, 2.2)	1.2 (0.63, 2.2)
	> 2.1–3.5	123	31	1.4 (0.8, 2.5)	1.5 (0.84, 2.8)
	> 3.5	113	42	2.2 (1.2, 3.9)	2.2 (1.2, 4.0)
MCNP per log10 IQR		494	128	1.3 (1.0, 1.6)	1.3 (1.0, 1.7)
Abbreviations: IQR, interquartile range; ref, reference. aAdjusted for urine SG, sex, parental asthma, and household income.					

Sex modified the association between ΣDEHP (in quartiles) and current asthma (overall *p*_interaction_ = 0.03, and *p* < 0.05 for each of the quartiles). The aORs for current asthma in girls for the three highest concentration categories of ΣDEHP were 3.9 (95% CI: 1.2, 12.5), 5.9 (95% CI: 1.8, 19.2), and 4.6 (95% CI: 1.3, 16.3), *p*_trend_ = 0.04. The aORs for current asthma in boys by quartile concentrations of ΣDEHP were 0.54 (95% CI: 0.25, 1.19), 0.68 (95% CI: 0.31, 1.48), and 1.07 (95% CI: 0.50, 2.3) [see Supplemental Material, Figure S3 (http://dx.doi.org/10.1289/ehp.1205256)]. When ΣDEHP was used as a continuous variable in the model, no sex modification between ΣDEHP and current asthma was observed (*p*_interaction_ = 0.2), aOR of 1.2 (95% CI: 0.82, 1.83) per IQR log_10_ unit increase in ΣDEHP for girls and 0.87 (95% CI: 0.62, 1.2) for boys.

Current asthma was not statistically significantly associated with urinary concentrations of MEP, MnBP, MiBP, MBzP, or MCPP (Table 3). The ORs for current asthma increased by quartiles for ΣDEHP and Σhigh-MWP (Table 4), but the individual estimates were not statistically significant.

**Table 4:** Crude and adjusted odds ratios of current asthma per concentration quartile and per log10 IQR increase in ΣDEHP, Σlow-MWP, and Σhigh-MWP.

Phthalate metabolite sum	Concentration (µmol/L)	Noncases (n)	Cases (n)	OR (95% CI)	aORa (95% CI)
ΣDEHPb In quartiles	≤ 0.50 (ref)	123	31	1	1
	> 0.50–0.79	129	27	0.90 (0.50, 1.6)	1.1 (0.65, 1.9)
	> 0.79–1.23	122	34	1.3 (0.69, 2.3)	1.4 (0.75, 2.6)
	> 1.23	120	36	1.4 (0.75, 2.7)	1.6 (0.83, 3.2)
ΣDEHP per log10 IQR		494	128	0.96 (0.73, 1.3)	0.99 (0.75, 1.3)
Σlow-MWPc in quartiles	≤ 0.85 (ref)	121	34	1	1
	> 0.85–1.26	126	28	0.81 (0.46, 1.4)	0.83 (0.46, 1.5)
	> 1.26–1.97	121	35	1.1 (0.60, 1.9)	1.3 (0.71, 2.3)
	> 1.97	125	31	0.92 (0.51, 1.7)	0.95 (0.51, 1.8)
Σlow-MWP per log10 IQR		494	128	0.98 (0.76, 1.3)	1.0 (0.77, 1.3)
Σhigh-MWPd in quartiles	≤ 0.66 (ref)	125	29	1	1
	> 0.66–1.0	126	29	1.1 (0.61, 2.0)	1.2 (0.65, 2.2)
	> 1.0–1.56	123	32	1.3 (0.72, 2.5)	1.4 (0.72, 2.6)
	> 1.56	118	38	1.8 (0.92, 3.4)	1,8 (0.92, 3.6)
Σhigh-MWP per log10 IQR		494	128	1.0 (0.76, 1.3)	1.0 (0.77, 1.4)
Abbreviations: IQR, interquartile range; ref, reference. aAdjusted for urine SG, sex, parental asthma, and household income. b∑DEHP: MEHP, MECPP, MEHHP, and MEOHP. c∑Low-MWP: MEP, MnBP, and MiBP. d∑High-MWP: MBzP, MCNP, MCOP, MCPP, MEHP, MECPP, MEHHP, and MEOHP.					

## Discussion

The body burden of phthalates as determined by urinary concentrations of their metabolites was measurable in all study subjects in this Norwegian population in 2001–2004. Girls had higher urinary concentrations of the low-MWP metabolites than boys. We found associations between current asthma and the highest quartiles of MCOP and MCNP, metabolites of diisononyl phthalate (DINP) and DIDP, respectively, but not with any of the other phthalate metabolites evaluated in this study.

The present study population had, by design, a high prevalence of current asthma, and is not representative of the general population of Norwegian 10-year-old children. However, the concentrations of most phthalate metabolites were similar to those in previous reports for children in Europe and the United States. The urinary concentrations of the four DEHP metabolites were comparable to concentrations reported for children 6–11 years old from the 2001–2004 U.S. National Health and Nutrition Examination Survey (NHANES) (CDC 2012), and MCOP and MCNP levels were comparable to those in adolescents in NHANES 2005–2006 (Calafat et al. 2011). DEHP, MiBP, MnBP, and MCOP concentrations in the Norwegian children were comparable to levels reported for German children (Becker et al. 2009; Koch et al. 2007, 2011). However, caution should be taken when comparing studies, as differences in use of spot (NHANES) versus first morning voids (the present study), may affect the levels of phthalate metabolites in urine (Preau et al. 2010).

In the present study, the concentration of MBzP was associated with low household income, low maternal education level, and a BMI > 85th percentile—characteristics that are highly associated with each other. The reasons for a higher concentration of MBzP within these groups are unknown, but may be related to differences in diet, housing characteristics, or other factors. The concentration of MEP was higher in children of parents who smoked at home and in children whose mothers had the lowest level of education. Higher MEP concentrations were previously reported among male smokers compared with male nonsmokers (Duty et al. 2005).

Girls had higher urinary concentrations of the low-MWP metabolites MnBP and MEP than did boys. Similar differences have been reported for adults (Silva et al. 2004) and for German students (age range, 20–29 years) for MnBP and MiBP (Wittassek et al. 2007b), whereas among healthy Danish children and adolescents (6–21 years of age), boys had significantly higher levels of MiBP and MnBP than girls (Frederiksen et al. 2011). In one previous study that measured four phthalates in particulate matter in indoor air samples from dwellings, schools, and kindergartens in Norway, di-*n*-butyl phthalate (DnBP) concentrations were higher in all locations compared with butylbenzyl phthalate (BBzP), DEHP, and dicyclohexyl phthalate (Rakkestad et al. 2007). Furthermore, significant correlations between personal air levels of DEP, DnBP, and BBzP and urinary concentrations of their corresponding metabolites have been reported (Adibi et al. 2003), suggesting that inhalation may be an important pathway of exposure to low-MWP. Reported sex differences in MEP concentrations in adults (Silva et al. 2004) have usually been explained by the use of cosmetics and body-care products, and perfume use has been suggested to be a significant source of DEP exposure in women (Just et al. 2010). This relation could however not be ascertained in the present study as use of personal care products was not assessed.

The metabolites MCOP and MCNP from the high-MWP DINP and DIDP, respectively, were modestly associated with current asthma in the present study. The associations were statistically significant only for the highest concentrations of MCOP and MCNP, but with a monotonic increasing trend by quartiles of MCOP and MCNP. In toxicological studies, DINP has been found to affect reproductive outcomes and the development of the male reproductive tract, and DIDP to produce rodent liver effects (Cho et al. 2008)—but these effects are observed mainly with exposure levels that are much higher than estimated human exposure. Experimental studies of DIDP and DINP with a focus on respiratory outcomes are so far lacking. Given the paucity of the data, it is also difficult to speculate about why the association between concentrations of MCNP and current asthma appears to be strongest among the nonsensitized children in our study. The strong correlation between MCOP and MCNP suggests similar sources of exposure to the parent compounds, which are both used primarily as plasticizers of PVC and may be used in flooring, wall coverings, building materials, heat-resistant electrical cords, car interiors, and toys. Previous studies have reported associations between exposure to PVC-containing interior surface materials (Jaakkola et al. 1999, 2006; Larsson et al. 2010) and high-MWP in dust with asthma and respiratory outcomes (Bornehag et al. 2004; Kolarik et al. 2008), but DINP and DIDP have usually not been measured in environmental samples.

Previous studies have reported associations between house dust concentrations of DEHP and asthma and wheeze in children (Bornehag et al. 2004; Kolarik et al. 2008), but none have reported evidence of differences by sex. However, due to the small numbers of observations after stratifying by sex, estimates were imprecise, increasing the uncertainty about the accuracy of the point estimates. In one early publication based on the 2-year follow-up of the children in the ECA study, DEHP was identified as the major source of phthalate exposure, both in house dust and particulate matter (Oie et al. 1997). DEHP, which was the most commonly used phthalate plasticizer for many years, is now being replaced by DINP and DIDP (Wittassek et al. 2011).

When assessing associations between phthalate biomarkers and respiratory outcomes, one concern is individuals who receive pulmonary therapy through PVC tubing that may contain DEHP (Tickner et al. 2001). However, very few children in the ECA study had severe asthma (10 of 154 with current asthma in the 10-year follow-up) (Lang et al. 2008), and the use of asthma medication as well as hospitalizations have been thoroughly reported in the parental interview at the 10-year follow-up investigation. We did not find any statistically significant associations by ANOVA between SG-adjusted phthalate metabolite concentrations and use of asthma medication (leukotriene agonists, β-2 agonists, and inhaled corticosteroids) during the 14 days before urine collection (results not shown). Although use of asthma medication did not explain the association with current asthma, the possibility of reverse causation cannot be ruled out given the cross-sectional design of this study. Although DIDP and DINP are used mainly as plasticizers of PVC—highlighted as a possible explanation for the associations reported between high-MWP in indoor dust and asthma (Jaakkola and Knight 2008)—most of the body burden of high-MWP metabolites is likely to be derived from contaminated foods (Schettler 2006). We cannot exclude the possibility that differences in phthalate metabolite levels are caused by differences in sources of exposure, such as diet and personal care products.

In the present study, we measured the oxidative metabolites MECPP, MEHHP, and MEOHP from DEHP and the oxidative metabolites MCOP and MCNP from DINP and DIDP, respectively. The oxidized phthalate metabolites are not susceptible to external contamination, and have longer half-lives than the monoesters and are therefore suggested to be suitable for capturing the average background exposure. Our measurements are based on a single urine sample only, and although spot urine samples and 24-hr urine samples produced comparable estimates of daily DEHP intake (Wittassek et al. 2007a), and a single urine measurement may be predictive of short-term exposure (Hoppin et al. 2002), the short biological half-life of phthalates make measurements based on single urine samples less representative of exposure over longer intervals (Hauser et al. 2004; Preau et al. 2010). We could not evaluate the possibility of metabolites degradation during long-term storage of our samples. However, phthalate metabolites are considered chemically stable and the high correlation between the concentrations of DEHP metabolites is a good indicator of stability (Baird et al. 2010). Whereas other studies to date have looked at a few high-MWPs (mainly DEHP and BBzP), we assessed a wide selection of high-MWP in association with current asthma in children.

## Conclusions

Urinary concentrations of phthalate metabolites in Norwegian children were comparable to concentrations reported from children in other European countries and in the United States. Current asthma was modestly associated with urinary concentrations of DINP and DIDP metabolites. Although our findings add to the increasing literature on high-MWP exposure and asthma, the results of this study should be interpreted with caution due the cross-sectional design and the short half-life of the phthalate metabolites.

## Supplemental Material

(164 KB) PDF
