# Supplementary material for: Urinary Biomarkers for Phthalates Associated with Asthma in Norwegian Children
Source: Environ Health Perspect. 2012 Nov 16;121(2):251–6. doi: 10.1289/ehp.1205256 (PMC3569683; doi:10.1289/ehp.1205256)
Supplement: (164 KB) PDF [file ehp.1205256.s001.pdf]

**Supplemental Material**

**Urinary Biomarkers for Phthalates Associated with Asthma in Norwegian Children**

Randi J. Bertelsen, Karin C. Lødrup Carlsen, Antonia M. Calafat, Jane A. Hoppin, Geir Håland, Petter Mowinckel, Kai-Håkon Carlsen, Martinus Løvik

**Table of contents:**

Page 2. **Figure S1:** Flow-chart of the 623 children within the Environment and Childhood Asthma study with urine analysed for phthalate metabolites

Page 3. **Table S1:** Comparison of characteristics between 623 children included in the present study and 396 non-included children from the 10-year follow-up.

Page 4. **Table S2:** Demography at birth of 623 children with urine analysed for phthalate metabolites at age 10 compared to the remainder of the birth cohort (n=3131).

Page 5. **Figure S2:** Directed acyclic graph (DAG) for evaluation of covariate selection in the analyses of phthalates and current asthma.

Page 6. **Table S3:** Spearman rank correlation coefficient, $r_s$, for correlation between the individual phthalate metabolites

Page 7. **Table S4:** Geometric mean (GM) and 95% confidence interval (CI) for specific gravity adjusted urinary concentrations of individual phthalate metabolites (µg/L) and phthalate sums (µmol/L) for girls and boys (p-value for one-way ANOVA)

Page 8. **Table S5:** Adjusted odds ratio for current asthma (n=52) in children *without* allergic sensitization (n=392) and for current asthma (n=70) in children *with* allergic sensitization (n=210) per $\log_{10}$ IQR unit increase in urinary concentration of phthalate metabolites.

Page 9. **Figure S3:** Adjusted oddsratio for current asthma for girls (white dots) and boys (black dots) by quartiles of ∑DEHP concentration (in µmol/L) adjusted for urine specific gravity, parental asthma, and household

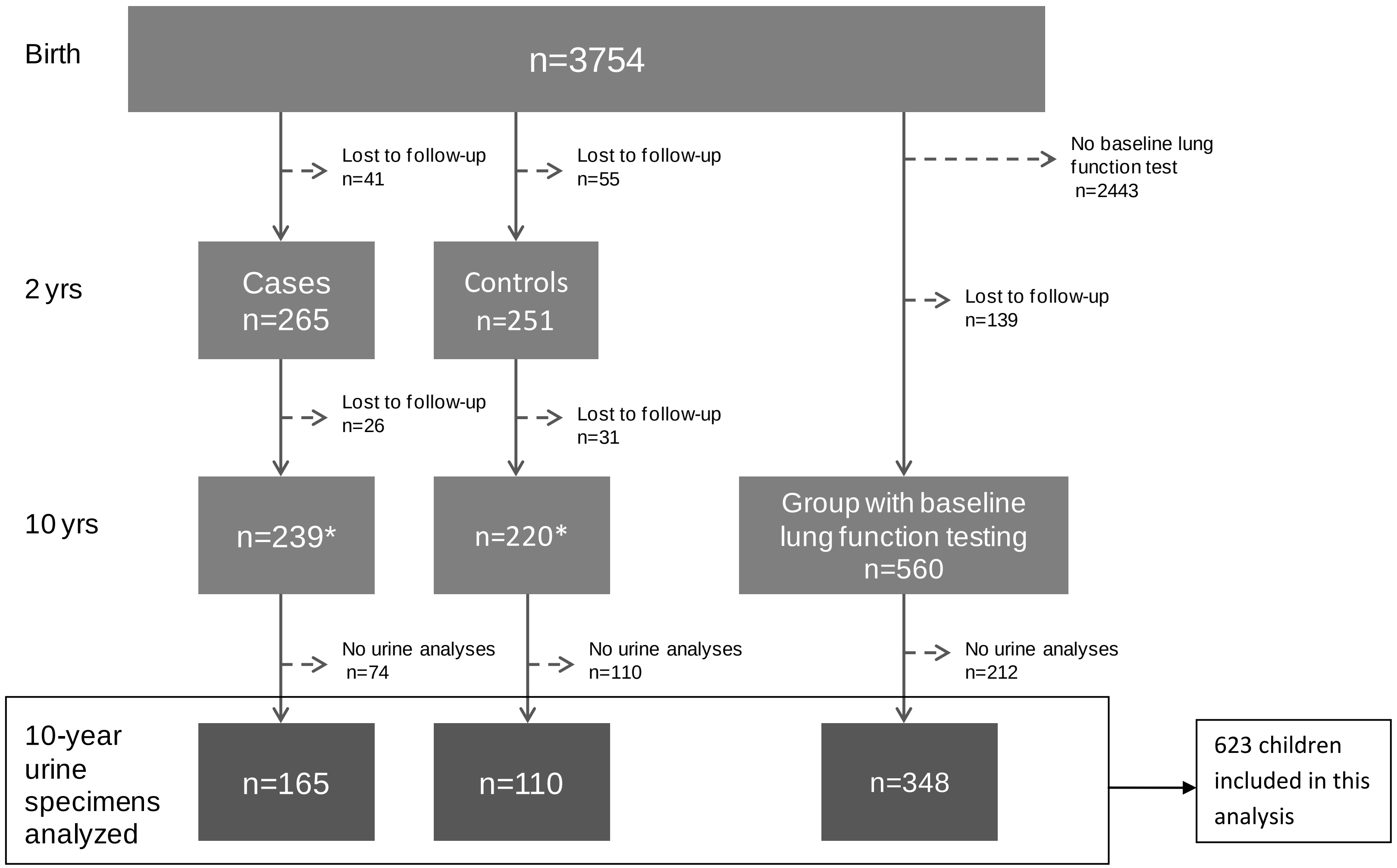


*Among the children followed up at age 10, 63 of the cases and 73 of the controls had had lung function tests at birth

**Figure S1:** Flow-chart of the 623 children within the Environment and Childhood Asthma study with urine analyzed for phthalate metabolites

**Table S1:** Comparisons of characteristics between 623 children included in the present study and the 396 *non-included* children from the 10-year follow-up. Percent of characteristics within each group, except for age (in years) reported by median (min-max)

| **Characteristics** | **% of n=623** | **% of n=396** | **p-value** |
|---|---|---|---|
| **SUBJECTS** | | | |
| Age [yrs, median (min-max)] | 10.7 (8.8-12.5) | 10.8 (9.0-12.5) | 0.001 |
| Boys | 53 | 56 | 0.4 |
| [a]BMI ≥ 85$^{th}$ percentile | 16 | 18 | 0.3 |
| Firstborn | 49 | 56 | 0.02 |
| Skin prick test (SPT) positive | 26 | 34 | 0.02 |
| sIgE > 0.35 kU/L | 33 | 39 | 0.04 |
| Either SPT positive or sIgE > 0.35 kU/L | 35 | 43 | 0.02 |
| Current rhinitis | 26 | 28 | 0.06 |
| Current asthma | 21 | 7 | <0.001 |
| Current eczema | 23 | 18 | 0.05 |
| **PARENTS** | | | |
| Parental asthma *or* rhinoconjuctivitis at child's birth | 36 | 37 | 0.8 |
| Maternal education, years | | | |
| ≤ 12 | 47 | 53 | 0.1 |
| 13-16 | 31 | 27 | |
| ≥ 17 | 22 | 20 | |
| Annual household income (in 1000 NOK[b]) | | | |
| <350 | 13 | 20 | 0.2 |
| > 350 - 560 | 28 | 24 | |
| > 560 - 750 | 30 | 26 | |
| > 750 | 30 | 30 | |

[a]age- and gender adjusted BMI; [b]NOK=Norwegian krone

**Table S2:** Demography at birth of 623 children with urine analysed for phthalate metabolites at age 10 compared to the remainder of the birth cohort (n=3131). Reported as percent of characteristics within each group

| Characteristics | % of included n=623 | % of non-included n=3131 | p-value |
|---|---|---|---|
| Gender (boys) | 53 | 52 | 0.5 |
| Parental asthma[a] | 14 | 12 | 0.1 |
| Parental rhinoconjunctivitis[a] | 31 | 27 | 0.08 |
| Parental atopic eczema[a] | 30 | 28 | 0.5 |
| Maternal smoking in pregnancy | | | |
| No | 75 | 76 | |
| Occasionally | 10 | 9 | |
| Daily | 15 | 15 | 0.9 |
| Firstborn | 49 | 56 | <0.001 |
| Pets at birth | 23 | 23 | 0.9 |
| Annual household income (in 1000 NOK[b]) | | | |
| ≤299 | 24 | 31 | |
| 300 -499 | 60 | 49 | |
| ≥ 500 | 16 | 20 | <0.001 |
| Maternal education | | | |
| ≤ 12 years | 47 | 47 | |
| 13-16 | 31 | 30 | |
| ≥ 17 years | 22 | 24 | 0.5 |
| Parents living together | 95 | 94 | 0.3 |

[a]Parental allergic diseases are reported as the presence of disease in mother, father or both;
[b]NOK=Norwegian krone

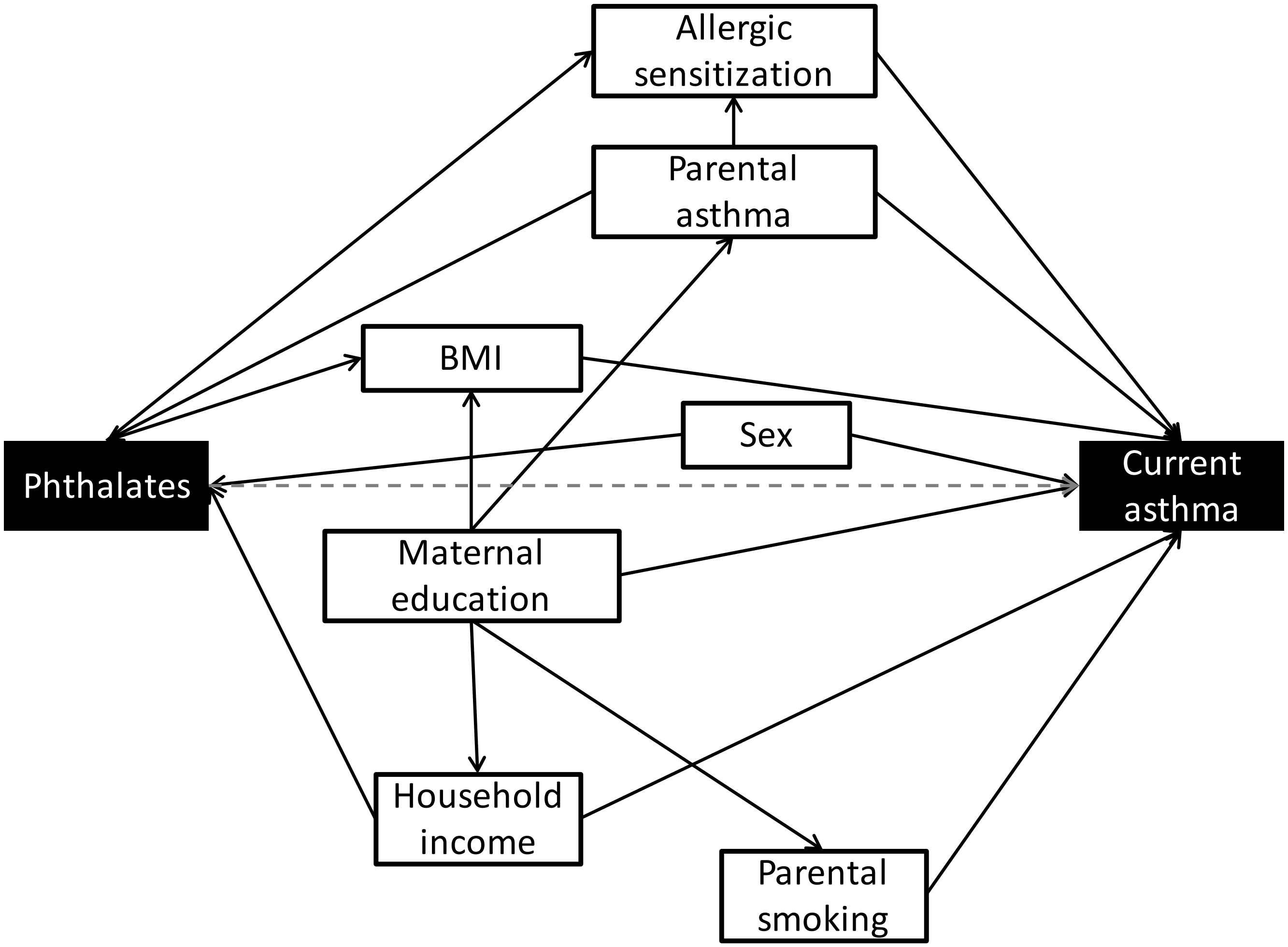


**Figure S2:** Directed acyclic graph (DAG) for evaluation of covariate selection in the analyses of phthalates and current asthma. The minimial adjustment set for estimating the total effect of phthalates on asthma was according to the dagitty (http://www.dagitty. net/dags/html): sex, household income, and parental asthma

**Table S3:** Spearman rank correlation coefficient, $r_s$, for correlation between the individual phthalate metabolites

| | **MnBP** | **MiBP** | **MBzP** | **MCPP** | **MEHP** | **MEOHP** | **MEHHP** | **MECPP** | **MCOP** | **MCNP** |
|---|---|---|---|---|---|---|---|---|---|---|
| **MEP** | 0.45 | 0.35 | 0.39 | 0.31 | 0.19 | 0.34 | 0.32 | 0.33 | 0.27 | 0.23 |
| **MnBP** | | 0.64 | 0.55 | 0.52 | 0.34 | 0.51 | 0.48 | 0.48 | 0.36 | 0.32 |
| **MiBP** | | | 0.50 | 0.44 | 0.26 | 0.55 | 0.51 | 0.50 | 0.39 | 0.33 |
| **MBzP** | | | | 0.47 | 0.30 | 0.48 | 0.46 | 0.47 | 0.41 | 0.38 |
| **MCPP** | | | | | 0.29 | 0.61 | 0.61 | 0.58 | 0.59 | 0.54 |
| **MEHP** | | | | | | 0.62 | 0.62 | 0.58 | 0.27 | 0.25 |
| **MEOHP** | | | | | | | 0.98 | 0.94 | 0.51 | 0.44 |
| **MEHHP** | | | | | | | | 0.93 | 0.50 | 0.43 |
| **MECPP** | | | | | | | | | 0.52 | 0.47 |
| **MCOP** | | | | | | | | | | 0.75 |

**Table S4:** Geometric mean (GM) and 95% confidence interval (CI) for specific gravity adjusted urinary concentrations of individual phthalate metabolites (µg/L) and phthalate sums (µmol/L) for girls and boys (p-value for one-way ANOVA)

| Phthalate | Girls GM (95% CI) | Boys GM (95% CI) | p |
|---|---|---|---|
| *Individual phthalate metabolites (parent phthalate)* | | | |
| MEP (DEP) | 77 (69, 85) | 55 (50, 60) | <0.001 |
| MnBP (DnBP) | 161 (150, 172) | 135 (127, 144) | <0.001 |
| MiBP (DiBP) | 61 (56, 66) | 54 (50, 59) | 0.07 |
| MBzP (BBzP) | 34 (31, 37) | 31 (29, 34) | 0.3 |
| MCPP (DnOP) | 8.4 (7.7, 7.5) | 8.1 (7.5, 8.7) | 0.5 |
| MEHP (DEHP) | 8.0 (7.3, 8.7) | 8.4 (7.7, 9.1) | 0.4 |
| MEOHP (DEHP) | 52 (49, 56) | 53 (49, 57) | 0.9 |
| MEHHP (DEHP) | 83 (77, 89) | 84 (78, 90) | 0.8 |
| MECPP (DEHP) | 109 (101, 118) | 105 (97, 113) | 0.4 |
| MCOP (DINP) | 6.9 (6.4, 7.6) | 6.1 (5.6, 6.6) | 0.03 |
| MCNP (DIDP) | 2.5 (2.3, 2.7) | 2.2 (2.0, 2.4) | 0.09 |
| *Phthalate sums* | | | |
| ∑Low-MWP [a] | 1.6 (1.5, 1.7) | 1.3 (1.2, 1.4) | <0.001 |
| ∑High-MWP [b] | 1.1 (1.1, 1.2) | 1.1 (1.0, 1.2) | 0.5 |
| ∑DEHP [c] | 0.9 (0.8, 0.9) | 0.9 (0.8, 0.9) | 0.9 |

The MEP and MBzP concentrations have been multiplied by 0.66 and 0.72, respectively, to correct for the inadequate purity of the analytic standards used (Calafat, personal communication, 2012).

[a]∑Low-MWP: MEP, MnBP, and MiBP. [b]∑High-MWP: MBzP, MCNP, MCOP, MCPP, MEHP, MECPP, MEHHP, and MEOHP. [c]∑DEHP : MEHP, MECPP, MEHHP, and MEOHP.

**Table S5:** Adjusted odds ratio for current asthma (n=52) in children *without* allergic sensitization (n=392) and for current asthma (n=70) in children *with* allergic sensitization (n=210) per $log_{10}$ IQR unit increase in urinary concentration of phthalate metabolites. Adjusted for urine specific gravity, parental asthma and household income

| | **Allergically sensitized** | |
|---|---|---|
| **Phthalate metabolites** | **No (n=392) aOR (95% CI)** | **Yes (n=210) aOR (95% CI)** |
| MEP | 1.1 (0.75, 1.5) | 1.0 (0.69, 1.5) |
| MnBP | 0.98 (0.64, 1.5) | 0.76 (0.50, 1.2) |
| MiBP | 1.2 (0.77, 1.7) | 1.3 (0.86, 1.84) |
| MBzP | 1.2 (0.83, 1.8) | 1.1 (0.73, 1.8) |
| MCPP | 1.4 (0.87, 2.2) | 0.99 (0.67, 1.5) |
| MCOP | 1.4 (0.95, 2.1) | 1.4 (0.87, 2.1) |
| MCNP | 1.6 (1.1, 2.2) | 1.1 (0.77, 1.5) |
| **Metabolite sums** | | |
| ΣLow-MWP[a] | 1.1 (.075, 1.6) | 1.0 (0.68, 1.5) |
| ΣHigh-MWP[b] | 1.2 (0.79, 1.8) | 1.1 (0.67, 1.6) |
| ΣDEHP[c] | 1.2 (0.77, 1.7) | 0.99 (0.64, 1.5) |

[a]∑Low-MWP: MEP, MnBP, and MiBP. [b]∑High-MWP: MBzP, MCNP, MCOP, MCPP, MEHP, MECPP, MEHHP, and MEOHP. [c]∑DEHP : MEHP, MECPP, MEHHP, and MEOHP.

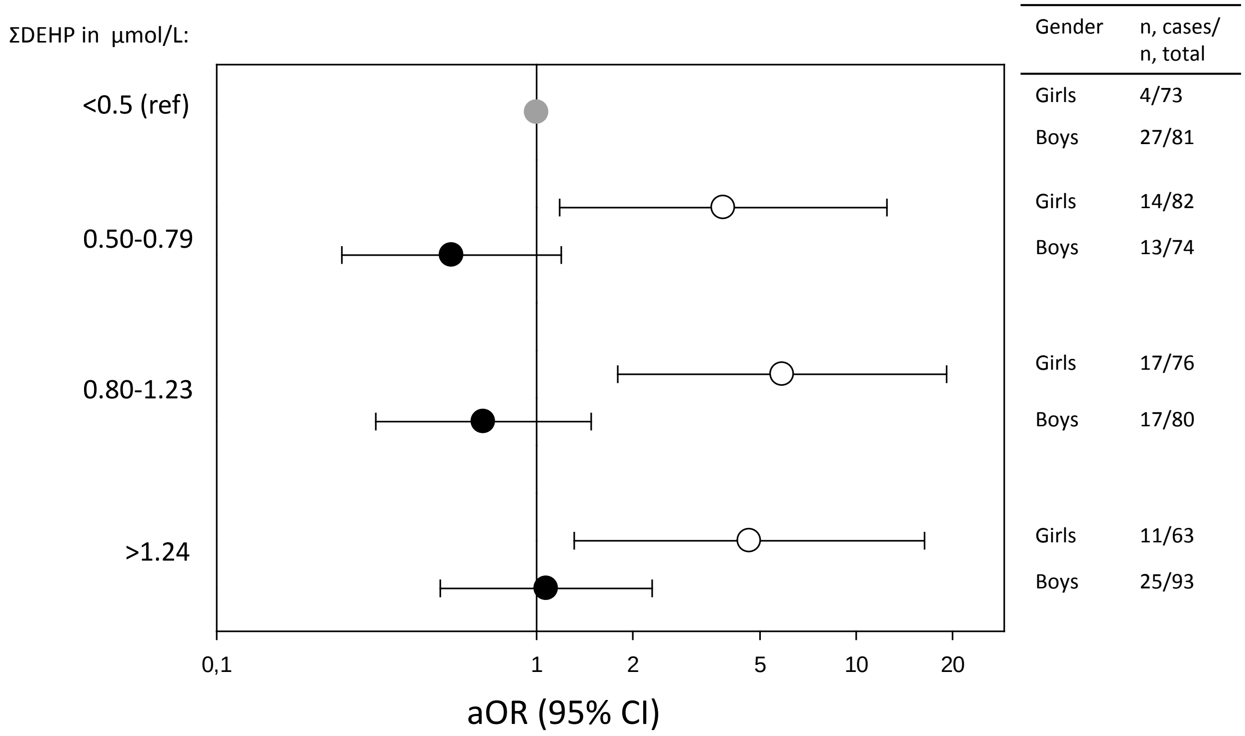


**Figure S3:** Adjusted oddsratio for current asthma for girls (white dots) and boys (black dots) by quartiles of ∑DEHP concentration (in µmol/L) adjusted for urine specific gravity, parental asthma, and household
